# Learning-related representational changes reveal dissociable integration and separation signatures in the hippocampus and prefrontal cortex

**DOI:** 10.1038/ncomms9151

**Published:** 2015-08-25

**Authors:** Margaret L. Schlichting, Jeanette A. Mumford, Alison R. Preston

**Affiliations:** 1 Center for Learning and Memory, The University of Texas at Austin, 1 University Station, C7000, Austin, Texas 78712, USA; 2 Department of Psychology, The University of Texas at Austin, 1 University Station, A8000, Austin, TX 78712, USA; 3 Center for Investigating Healthy Minds at the Waisman Center, University of Wisconsin-Madison, 1500 Highland Avenue, Suite S119, Madison, Wisconsin 53705-2280, USA; 4 Department of Neuroscience, The University of Texas at Austin, 1 University Station, C0920, Austin, TX 78712, USA

## Abstract

The episodic memory system enables accurate retrieval while maintaining flexibility by representing both specific episodes and generalizations across events. Although theories suggest that the hippocampus (HPC) is dedicated to represent specific episodes while the medial prefrontal cortex (MPFC) generalizes, other accounts posit that HPC can also integrate related memories. Here we use high-resolution functional magnetic resonance imaging in humans to examine how representations of memory elements change to either differentiate or generalize across related events. We show that while posterior HPC and anterior MPFC maintain distinct memories for individual events, anterior HPC and posterior MPFC integrate across memories. Integration is particularly likely for established memories versus those encoded simultaneously, highlighting the greater impact of prior knowledge on new encoding. We also show dissociable coding signatures in ventrolateral PFC, a region previously implicated in interference resolution. These data highlight how memory elements are represented to simultaneously promote generalization across memories and protect from interference.

## Abstract (web summary)

Our memory system maintains flexibility by representing both specific events as well as generalizations across events, yet the brain regions supporting each remain unknown. Here the authors reveal dissociable neural signatures of memory separation and integration in the hippocampus and prefrontal cortex.

## Graphical abstract

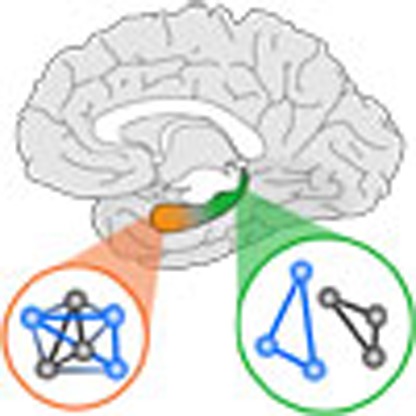

Our memory system faces the challenge of simultaneously representing both commonalities and idiosyncrasies across experiences. For instance, in some cases it is advantageous to generalize across trips to the supermarket, as when inferring where to find milk in a new store. However, doing so will not help you remember where you parked your car on today's grocery run. Intuitively, it seems that our brains maintain both separated memories that keep even highly similar events distinct and integrated memories that combine events. Yet, the circumstances and brain regions supporting each remain unknown.

The tension between separation and integration stems from the conceptualization of the hippocampus (HPC) as a fast-learning system that rapidly encodes pattern-separated memories. Under this model, even related events are coded by largely non-overlapping populations of HPC neurons^1^,^2^,^3^, a scheme thought to support detailed retrieval and protect from interference^1^. The complementary learning systems (CLS) framework additionally proposes a slow-learning neocortical system, which integrates memories over time^1^,^2^,^3^. The idea that HPC and neocortex store complementary traces has received empirical support from studies showing increasing reliance on the neocortex with consolidation^4^ as memories become schematized^5^.

However, HPC also performs pattern completion, which may lead to integration in some circumstances^1^,^2^,^6^,^7^,^8^. In particular, when experiences share features, HPC may retrieve the existing, related memory instead of separately encoding the new event^1^,^6^,^8^. Pattern completion during new memory formation would result in overlapping populations of HPC neurons coding related events^1^,^9^, termed integration. Rodent work has demonstrated the existence of integrated codes, in which shared features act as linking ‘nodes'^10^,^11^. Moreover, related signatures in humans have been associated with flexible behaviours such as novel inference^12^,^13^,^14^,^15^.

We taught participants overlapping AB and BC associations, with the B item shared between pairs. Participants later completed a surprise inference test, in which they related indirectly associated A and C items. Of critical interest was (1) how indirectly related A and C elements are coded in HPC, (2) how learning conditions may bias the HPC towards completion or separation, and (3) how HPC coding schemes under these learning conditions may relate to individual differences in HPC anatomy. We operationalize separation as neural representations for A and C becoming more dissimilar after learning, consistent with differentiation of neuronal codes for related AB and BC memories^16^. Integration via nodal coding is defined as learning-related increases in similarity of A and C due to their common association with node B.

Notably, both separated and integrated codes may support inference^1^,^14^,^17^. In the case of separation, memories for individual events would be retrieved and recombined through logical reasoning^14^ or via recurrent connections^17^. In contrast, integration would enable the AC inference to be extracted directly from the integrated trace^14^,^15^. We propose that these coding schemes are not mutually exclusive; rather, separation and integration may alternately support inference on an event-by-event basis. Structural features such as HPC volume may additionally influence the preferred coding strategy.

The present study aims to determine the learning conditions that bias the memory system towards pattern completion and integration over separation. One factor that may influence the neural representations recruited for a given pair of overlapping events is the status of the initial (AB) memory at the beginning of new related learning. Existing theories alternately predict that stronger^18^ or more recently encoded^19^,^20^ AB memories will promote pattern completion during a new, overlapping event. For instance, strong AB representations may promote pattern completion during BC, thereby allowing for the formation of overlapping traces^1^ and behavioural flexibility^18^. Consistent with this hypothesis, enhanced generalization performance has been reported for strong memories^13^,^18^,^21^,^22^,^23^,^24^. However, strong memories are not necessary for integration^25^,^26^, as related signatures have been observed after just a single AB experience^12^,^27^. One possible explanation for these findings stems from temporal context theories^19^,^20^, which predict that encoding overlapping events close in time leads to greater representational overlap. Mechanistically, this might result from an enhanced tendency to pattern complete to recent events, a prediction that also receives support from models of HPC function^1^. This framework suggests that having recently encoded AB associations during BC learning would result in more similar A and C representations.

Different coding strategies may also be preferred across HPC subregions. For example, rodent work has shown that while anterior HPC neurons respond similarly across related episodes, posterior HPC firing patterns are event-specific^28^. Moreover, the ability to retrieve details has been differentially related to HPC volumes across the long axis, with smaller anterior and larger posterior regions being associated with superior recollection across individuals^29^. These findings and others^30^ suggest dissociable functions along the HPC anterior–posterior axis, with anterior generalizing across events and posterior representing event details^29^. Despite the prominence of these theories, there is little empirical evidence as to how elements of overlapping events are coded in human HPC. Moreover, as past work on this topic has focused almost exclusively on HPC coding, it remains unknown how prefrontal (PFC) regions might represent overlapping memories. Both medial and lateral PFC have been implicated in overlapping encoding and successful inference, with engagement of medial PFC (MPFC) during learning^13^ and inferior frontal gyrus (IFG) during inference^12^ predicting performance. Accordingly, one theoretical framework^22^ suggests that while MPFC generalizes across events as they are experienced, IFG represents individual episodes for later recombination.

Here we use high-resolution functional magnetic resonance imaging (hr-fMRI) to investigate representational changes within HPC and PFC. We predicted that while posterior HPC might pattern separate indirectly related A and C items, anterior HPC would integrate. We also hypothesized a dissociation in PFC, with MPFC showing integration and IFG showing separation. Consistent with these predictions, we show that while posterior HPC and anterior MPFC separate related events, anterior HPC and posterior MPFC tend to integrate. Integration is particularly likely when related memories are pre-established relative to when they are learned simultaneously, suggesting that strong memories—although encoded more remotely—promote pattern completion during new learning. IFG also exhibits dissociable coding signatures that are modulated by learning condition. The present study moves beyond identifying regions activated during overlapping encoding to show how these regions simultaneously promote generalization and protect from interference.

## Results

### Behavioural performance

**Figure 1:**
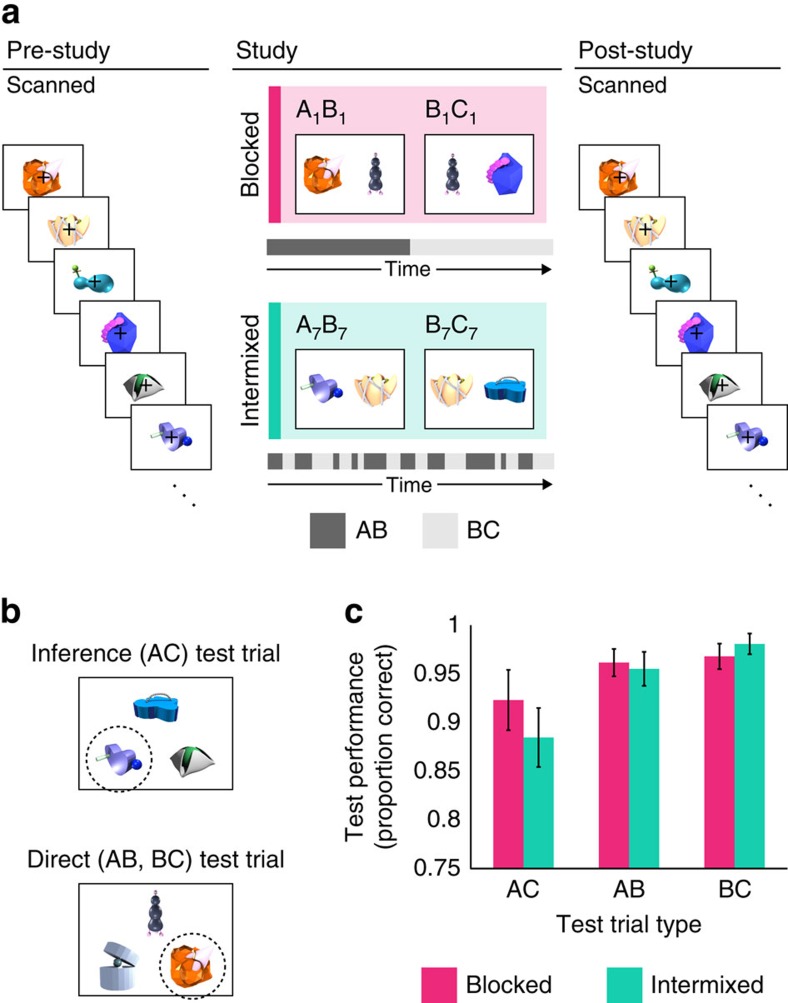
Paradigm overview and behavioural results. (**a**) During the study phase (middle), participants intentionally encoded pairs of novel objects. Half of the pairs were presented in a blocked manner (top, pink); half were intermixed (bottom, teal). Identical stimulus exposure phases occurred immediately before (left) and after (right) the study task during hr-fMRI scanning. Pre- and post-study exposure phases were used to obtain estimates of the neural patterns evoked by specific stimuli learned during the study phase. Trial timing and order were matched between pre- and post-study to avoid introducing unequal biases into the neural pattern estimates from the two phases. (**b**) After scanning, participants completed a two-alternative forced choice test for inference (top, tested first) and directly learned (bottom, tested second) associations. Participants selected which of the two choice stimuli (bottom of screen) was associated with the cue stimulus (top of screen). Correct answers are indicated with dashed circle (not shown to participants). (**c**) Performance as a function of test trial type and learning condition. Left bar pair, AC inference performance for blocked (pink; 33.3–100%, 92.3±3.1%) and intermixed (teal; 50–100%, 88.5±3.0%) triads. Middle bar pair, AB performance (blocked: 83.3–100%, 96.2±1.4%; intermixed: 66.7–100%, 95.5±1.7%). Right bar pair, BC performance (blocked: 83.3–100%, 96.8±1.3%; intermixed: 83.3–100%, 98.1±1.1%). Bar heights represent group means; error bars denote s.e.m. *N*=26 participants.

To assess the effects of learning experience on neural representations, each participant learned half of the overlapping pairs in a blocked and half in an intermixed manner (order counterbalanced across participants; Fig. 1a and Methods). One way in which these conditions differ is how established or recent the initial memory is on first encountering the overlapping pair. In the blocked learning condition, all 12 AB pair presentations occurred before any BC presentations. This would ensure that initial AB memories were established on first encountering the overlapping BC information, which might promote pattern completion to AB and integration. In the intermixed condition, overlapping pairs were encoded in alternation, such that initial AB memories were more recent (although weaker) when BC was first learned. As pattern completion also shows a bias towards recent information^1^, encoding AB and BC in close temporal proximity might also result in A–C similarity increases.

Memory for both directly learned (Fig. 1b) AB (collapsed across learning condition; range: 83.3–100%; mean±s.e.m.=95.8±1.3%; *t*_25_=34.52, *P*<1 × 10^−21^) and BC (91.7–100%; 97.4±0.8%; *t*_25_=61.67, *P*<1 × 10^−28^) pairs was above chance, indicating that participants had sufficient training on the premise associations. AC inference (Fig. 1b) was also above chance (58.3–100%; 90.4±2.3%; *t*_25_=17.24, *P*<1 × 10^−14^). Investigating performance as a function of learning condition (Fig. 1c), we observed only a significant main effect of test trial type (*F*_2,50_=6.65, *P*=0.003), driven by lower AC inference than direct pair (AB and BC) memory performance. There was no significant main effect of learning condition (*F*_1,50_=0.43, *P*=0.518) nor a test trial type × learning condition interaction (*F*_2,50_=0.96, *P*=0.390). Because there were no behavioural differences across conditions, we note that any neural differences are unlikely attributable to differences in performance.

### Dissociable coding signatures across the long axis of HPC

**Figure 2:**
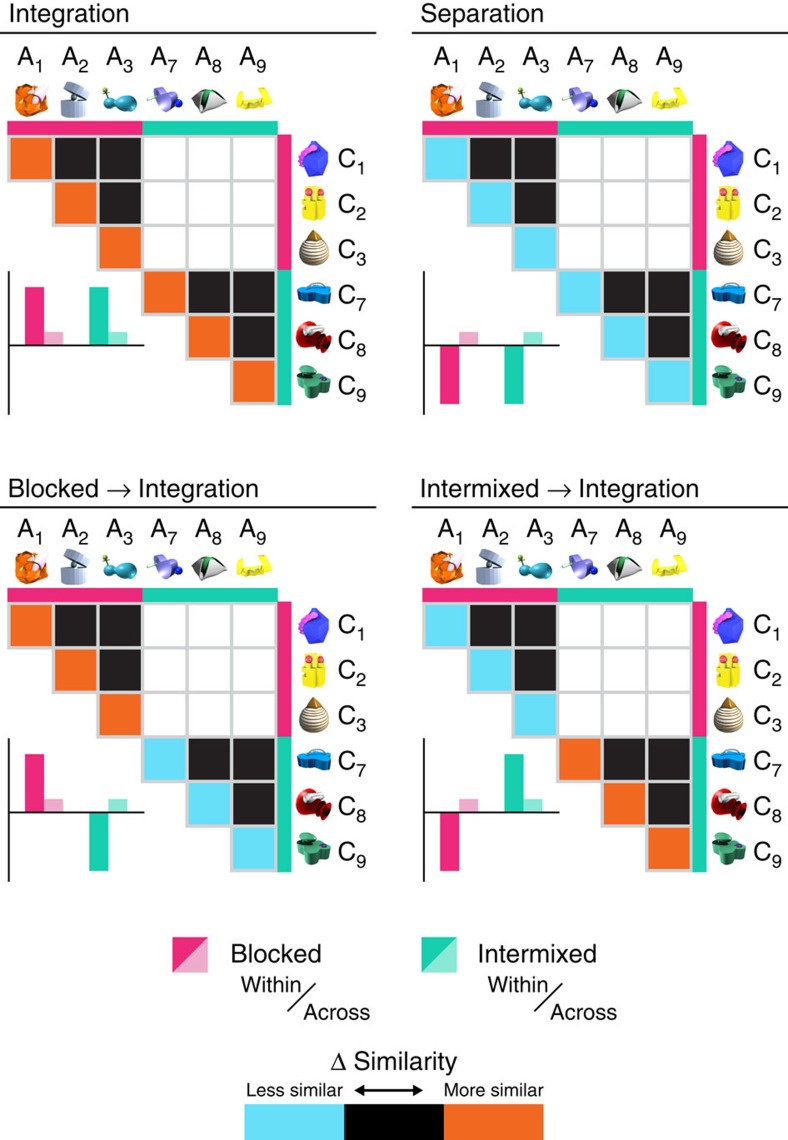
Predictions for RSA. Schematic depiction of RSA and predictions for a subset of six triads. Triads 1–3 were studied in a blocked manner (pink); triads 7–9 were intermixed (teal). For all matrices, the colour in each cell represents the predicted change in (Δ) RS between an A item (horizontal) and a C item (vertical) from pre- to post-study. Black cells indicate no change in similarity from pre- to post-study; orange cells indicate learning-related increases in similarity; blue cells indicate learning-related decreases in similarity. White cells represent comparisons across learning condition (for example, intermixed item A7 with blocked item C1), which were excluded from all analyses. Matrices depict predicted item similarities for regions showing integration for both blocked and intermixed learning (top left), separation for both blocked and intermixed learning (top right) and blocked → integration (bottom left), and intermixed → integration (bottom right) interactions with learning condition. Inset bar graphs show predicted average similarities across all within- (darker bars) and across-triad (lighter bars) comparisons. Integration and separation were operationalized as significantly greater (integration) or less (separation) within- than across-triad Δ RS.

Participants viewed brief presentations of single A, B, and C items during hr-fMRI scanning both before and after learning of overlapping AB and BC associations. We then investigated learning-related changes in the neural representations of indirectly related A and C items for evidence of separation and integration using a representational similarity analysis (RSA)^31^ searchlight constrained to anatomical regions of interest (ROIs). For all searchlights, we looked for voxels exhibiting one of the four hypothesized learning-related signatures in A–C RS (Fig. 2): (1) integration for both learning conditions; (2) separation for both learning conditions; (3) interaction, with blocked → integration (in line with the prediction that established AB memories would promote integration); and (4) interaction, with intermixed → integration (in line with the prediction that more recent AB memories would promote integration). All analyses were limited to those A and C items for which the AC inference judgment was correct during the test.

**Figure 3:**
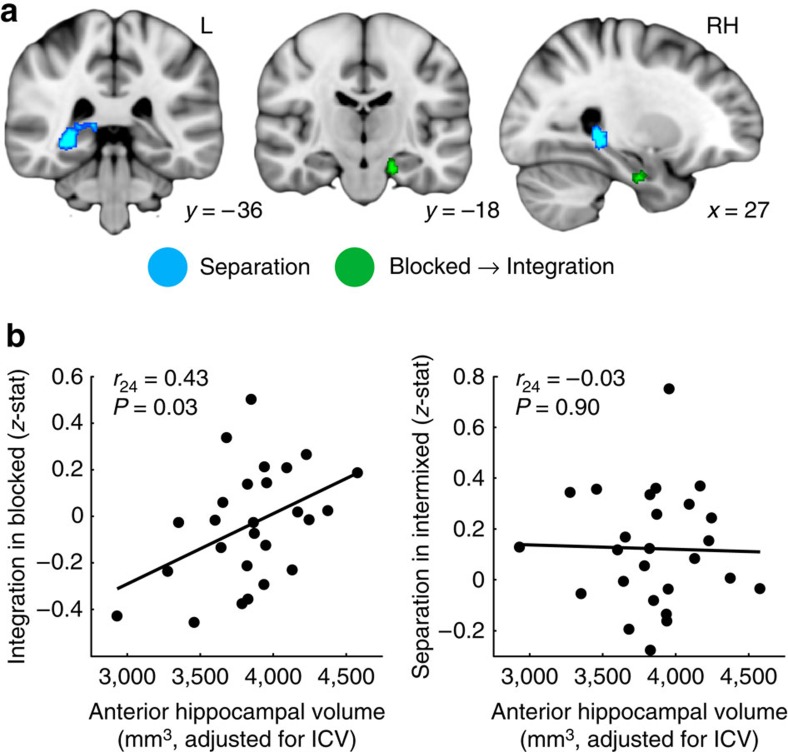
HPC representational similarity searchlight results. (**a**) HPC regions showing a significant main effect of separation (blue) or a blocked → integration interaction (green) displayed on the 1-mm MNI template brain. Clusters are significant after correction for multiple comparisons within anatomical HPC. Coordinates are in millimetres. (**b**) Across-participant relationship between anterior HPC volume (*x* axes) and evidence for integration in the blocked learning condition (*y* axis, left scatterplot; *r*=0.43, *P*=0.03) and separation in the intermixed learning condition (*y* axis, right scatterplot; *r*=−0.03, *P*=0.90) across the whole HPC. Statistics reflect Pearson correlations. *N*=26 participants. See also Supplementary Fig. 1.

Within HPC, we observed a right posterior cluster (Fig. 3a; cluster centre of gravity in the Montreal Neurological Institute (MNI) template coordinates (mm): *x*, *y*, *z*=28, −37, −1) that showed a significant main effect of separation, consistent with the proposed role of HPC in maintaining orthogonalized representations for overlapping events^2^. Follow-up analyses (Supplementary Methods and Supplementary Fig. 1) demonstrated significant separation (that is, larger decreases in RS for within- than across-triad) in this cluster for both blocked and intermixed triads when considered separately (both *P*<0.005). In contrast, two regions in anterior HPC (Fig. 3a; left: −17, −16, −21; right: 29, −11, −27) showed significant blocked → integration interactions (Fig. 2). In the left, this effect was driven by a significant separation effect for intermixed triads (*P*<0.0001), while the integration effect for blocked triads was at the trend level (*P*=0.081). Effects of both integration for blocked triads and separation for intermixed triads were significant in the right HPC cluster (both *P*<0.047). There were no significant learning-related changes in across-triad similarity for blocked or intermixed conditions in any HPC cluster (Bonferroni-corrected α-threshold for significance <0.005; all *P*>0.063; Supplementary Methods). No regions showed a significant main effect of integration or an intermixed → integration interaction.

### Anterior HPC volumes predict the degree of integration

We next assessed whether the degree of neural evidence for integration related to HPC structural measures. Prior work has linked HPC volumes to individual differences in memory performance^29^, yet the mechanism by which this relationship arises remains unknown. One possibility is that structural differences might influence the use of one representational scheme over another, thereby having an impact on behaviour. For instance, anterior HPC has been hypothesized to use its broad place fields to form generalized representations that span events and promote behavioural flexibility^29^,^32^. This region also shares the strongest anatomical connections with MPFC^33^, making it a good candidate region for explaining variability in the coding strategies employed across individuals. Thus, we hypothesized that more neural evidence for integration—particularly in the blocked learning condition—would relate across participants to volume of anterior HPC (defined here as HPC head; Supplementary Methods).

Anterior HPC volumes were positively related to the degree to which evidence for integration was observed in the blocked learning condition (*r*_24_=0.43, *P*=0.027; Fig. 3b, left), such that larger anterior HPC volumes were associated with more evidence for integration. There was no relationship between anterior HPC volume and separation in the intermixed condition (*r*_24_=−0.03, *P*=0.896; Fig. 3b, right). Posterior HPC segments (body and tail; see Supplementary Methods) did not relate to the degree of integration in the blocked condition (both |*r*_24_|<0.17, both *P*>0.414) or the degree of separation in the intermixed condition (both |*r*_24_|<0.06, both *P*>0.801). Moreover, when anterior (head) and posterior (body and tail) HPC volumes were simultaneously considered as independent variables in a multiple regression, only anterior HPC volume was a significant predictor of integration in the blocked condition (*β*=0.50, *P*=0.027; for posterior HPC body and tail volumes, both |*β*|<0.28, *P*>0.194). These findings highlight the relatively greater contribution of anterior HPC in integration. There was no relationship between volume of any subregion and the degree of separation in the intermixed condition (all |*β*|<0.05, all *P*>0.853) using multiple regression.

### Dissociable coding signatures in MPFC and IFG

**Figure 4:**
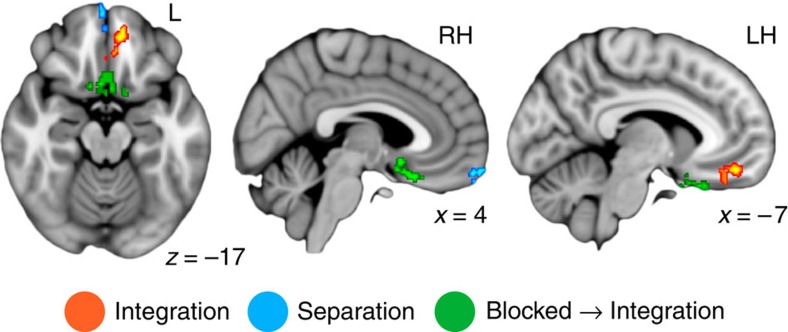
MPFC representational similarity searchlight results. MPFC regions showing a significant main effect of integration (orange), a main effect of separation (blue) or a blocked → integration interaction with learning condition (green) displayed on the 1-mm MNI template brain. Clusters are significant after correction for multiple comparisons within anatomical MPFC. Coordinates are in millimetres. *N*=26 participants. See also Supplementary Fig. 2.

Within MPFC, RSA searchlights revealed significant clusters for three of the four possible effects—main effects of integration, separation and a blocked → integration interaction, highlighting the functional hetereogeneity of this region (Fig. 4). A cluster in anterior MPFC exhibited a main effect of separation (1, 58, −20). Follow-up analyses revealed significant separation effects in this cluster for both blocked and intermixed learning conditions (both *P*<0.03). A slightly more posterior cluster demonstrated a main effect of integration (−8, 44, −17), with significant effects present in both learning conditions (both *P*<0.002). In the most posterior aspects of MPFC (that is, subgenual MPFC), two clusters demonstrated blocked → integration interactions with learning condition (3, 15, −17; −7, 18, −24), with integration for blocked and separation for intermixed triads. In both regions, effects of integration and separation were significant for blocked and intermixed learning conditions, respectively (Supplementary Methods and Supplementary Fig. 2; all *P*<0.033). No regions showed an intermixed → integration interaction.

**Figure 5:**
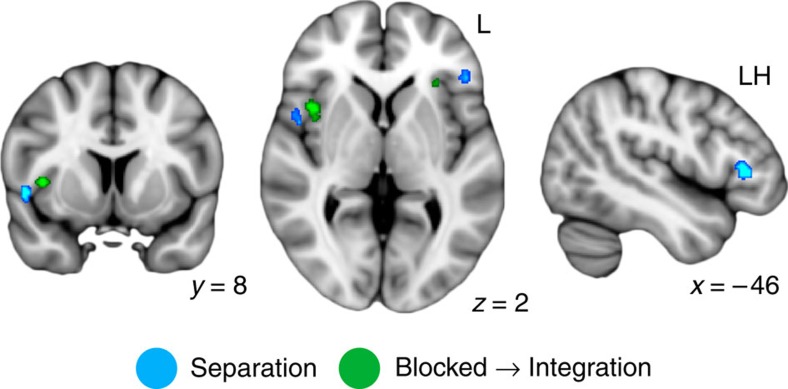
IFG representational similarity searchlight results. IFG regions showing a significant main effect of separation (blue) or a blocked → integration interaction with learning condition (green) displayed on the 1-mm MNI template brain. Clusters are significant after correction for multiple comparisons within anatomical IFG. Coordinates are in millimetres. *N*=26 participants. See also Supplementary Fig. 3.

The searchlight restricted to IFG revealed clusters showing main effects of separation and blocked → integration interactions with learning condition (Fig. 5). Main effects of separation were observed in an anterior left region (−47, 29, 6) and a more posterior right region (50, 6, −2). Significant separation was observed in both blocked and intermixed learning conditions for both regions (all *P*<0.020). Neighbouring regions in both the left and right hemispheres showed a significant interaction with learning condition (left: −29, 30, −2, right: 41, 10, 3; in the right hemisphere, this cluster extended into the insula). In both clusters, integration effects were significant for blocked and separation effects significant for intermixed triad types (Supplementary Methods and Supplementary Fig. 3; all *P*<0.039). There were no significant changes in across-triad similarity for blocked or intermixed conditions in any MPFC or IFG cluster (Bonferroni-corrected α-threshold for significance <0.005; all *P*>0.046; Supplementary Methods). No regions showed a main effect of integration or an intermixed → integration interaction.

### Whole-brain RSA searchlight results

RSA searchlights unrestricted to any particular region revealed a number of clusters showing significant effects of integration, separation and both interactions with learning condition (Supplementary Table 1). Just two regions showed main effects of integration, both in PFC. In contrast, numerous regions showed main effects of separation, including ventral occipitotemporal areas, temporal pole and insula. Still others showed interactions with learning condition. Regions including the midbrain, PFC, and higher-order visual regions showed evidence for integration in the blocked condition and separation for intermixed. In contrast, the precuneus, middle temporal gyrus and dorsal PFC showed the opposite pattern.

## Discussion

The present study combines hr-fMRI with RSA to identify the circumstances and brain regions that support integration over separation of related experiences. We show that the brain simultaneously maintains both integrated and separated representations of overlapping events, providing empirical evidence for the assumptions underlying computational memory theory^1^,^2^,^3^. These distinct representational coding schemes are dissociable across the brain and within subregions of HPC, MPFC, and IFG, highlighting the functional heterogeneity of these structures.

Specifically, within our *a priori* defined ROIs, we show that posterior HPC, bilateral IFG, and anterior MPFC evidence separation of overlapping events, with indirectly related A and C items becoming less similar to one another after learning. In contrast, mid-MPFC demonstrates integration of A and C. In other regions—anterior HPC, posterior MPFC, and more medial aspects of bilateral IFG—these representational changes are modulated by the manner in which the overlapping events occur, with blocked learning promoting integration. Notably, neither neural codes nor behaviour were modulated by differences in the order of blocked versus intermixed learning (Supplementary Methods). These findings are consistent with computational frameworks^1^ that suggest that HPC pattern completion during new learning will be more likely when initial memories are well established. We find that temporal proximity (that is, recency) on its own is not sufficient for integration^25^. While high levels of neural similarity have been shown for items experienced adjacent in time^34^, our results further demonstrate that such codes do not require temporal proximity; rather, having more established (that is, stronger) existing related memories at the time of first overlapping experience may be an additional factor promoting neural similarity.

By quantifying representational change at the level of specific elements^34^, we provide empirical evidence for a functional dissociation across the HPC axis. We demonstrate that posterior HPC separates across both learning conditions, with elements from overlapping memories being represented as more distinct than unrelated elements. This result is consistent with the view that posterior HPC individuates events from other, often highly similar, memories^22^,^29^,^35^. For instance, recent human fMRI work^36^ demonstrated lower RS in posterior HPC between segments of overlapping sequences, suggesting that this region disambiguates related events. However, as neural patterns were measured during sequence viewing and collapsed across items in that study, how the representations of specific elements within the sequences changed as a function of experience could not be determined. The present study builds on this existing literature to show that indirectly related memory elements are coded as distinct in HPC. Recent work has shown that such distinct representations may arise through an active differentiation process in HPC by which neural overlap among competing memories is eliminated^16^,^34^, particularly when overlapping events are studied and retrieved in alternation^36^. A similar mechanism may be at play in the present study, perhaps explaining the tendency for both anterior and posterior HPCs to take on distinct A–C representations in the intermixed condition.

In contrast to posterior HPC, anterior HPC (particularly on the right) integrates in the blocked learning condition. Prior work has suggested that this region forms generalized representations^22^,^29^, processes relational information^37^,^38^,^39^, and combines information across episodes^40^,^41^,^42^, typically on the basis of greater engagement during tasks that require consideration of multiple episodes. The present findings extend our current understanding to demonstrate *how* anterior HPC contributes—by adopting similar neural codes for indirectly related elements of experience. Interestingly, we also demonstrate a positive relationship between neural evidence for integration in the blocked condition and anterior HPC volumes, such that individuals with larger anterior HPC show more integration. This result may appear to contradict prior work, which has demonstrated superior memory for individuals with smaller anterior HPC^29^,^30^,^43^. One possible explanation that might reconcile these findings is that integration is harmful for performance as assessed by standard memory tasks. Specifically, if a memory task requires retrieval of episodic details, it may be more advantageous to separate rather than integrate, as integrated representations may code the commonalities across experiences while losing the specifics. Thus, it is possible that larger anterior HPC (that is, more integration) would be associated with superior performance on a task tapping generalization ability, while smaller anterior HPC would be more beneficial when a pattern separation scheme—supporting retrieval of specific episodic details—is required. The positive relationship between anterior HPC volume and integration may also seem counterintuitive from a mechanistic perspective, as smaller anterior HPC should have fewer neurons and thus may result in more overlap among memory traces. While the present data cannot provide a definitive reason for the observed association, one speculation is that larger HPC heads have more anatomical connections with MPFC, thereby biasing the system to integrate (see below for additional discussion related to this point). Alternatively, the distribution of HPC subfields may vary as a function of overall anterior HPC size, perhaps differentially biasing the overall response towards pattern completion or separation^7^,^44^.

The specificity of the integration signatures to the blocked learning condition is consistent with schema theory^18^ and modelling frameworks^1^, which suggest that more robust pattern completion of established memories will promote representational overlap. Having pre-established AB memories at the time of first BC encoding increases the likelihood that the overlapping B item will cue the previous (AB) memory. In contrast, the AB memory will be weaker during the first presentation of BC in the intermixed condition, thus biasing the system towards separation. More broadly, these findings corroborate a host of work, suggesting that strong initial memories can serve as a foundation on which new information is encoded^21^,^24^,^45^,^46^, further suggesting that such mechanisms might depend specifically on anterior HPC and its interactions with MPFC.

Our data suggest that, similar to HPC, MPFC exhibits functional differentiation along its anterior–posterior axis, with the most anterior aspects showing the separation of overlapping memories. One possibility is that anterior MPFC performs a more general mnemonic function, processing individual memories irrespective of their relationships to one another. In contrast, we observed evidence for integration in posterior MPFC. This finding builds on previous studies implicating this region in generalizing across events^47^,^48^,^49^; here we show that similar neural codes emerge in posterior MPFC for relationships that span experiences. In mid-MPFC, this pattern is true irrespective of learning condition. In contrast, subgenual MPFC exhibits integration for blocked but not intermixed learning conditions, mirroring the results in anterior HPC. While the neural measures reported here are not based on any measure of functional connectivity *per se*, one possible interpretation of this parallel is that subgenual MPFC and anterior HPC work in concert to form and store integrated representations. This notion is consistent with prior theoretical^22^,^47^ and empirical research, including work demonstrating the direct anatomical connections between subgenual MPFC and anterior HPC^33^. Collectively, these findings illuminate the mechanisms by which MPFC contributes to overlapping encoding and suggest important functional distinctions within this large region.

In contrast to MPFC, previous research has shown that IFG engagement during inference—but not during the preceding learning phase—predicts performance^12^, consistent with its proposed role in actively maintaining distinct individual event memories^50^,^51^ that may be recombined to address novel judgments. Here we identify bilateral IFG regions that show both separation and a blocked → integration interaction. In the left hemisphere, these regions are both in mid-IFG, corresponding approximately to pars triangularis/Brodmann area (BA) 45. This region has been widely implicated in resolving competition among similar alternatives and protecting from interference^50^,^51^. Our findings show that this region exhibits separated representational codes—perfectly suited for interference resolution. We also show a similar pattern in the right hemisphere in slightly more posterior regions (posterior-IFG or pars opercularis/BA 44). Thus, we suggest that both right and left IFG are involved in active maintenance of separate representations to resolve interference among related memories. Although right IFG has most often been implicated in inhibiting motor responses^52^, other work has demonstrated that right posterior-IFG is also sensitive to relational integration and interference resolution demands^53^. However, how our observed separation relates to the more widely demonstrated roles of right IFG^52^ remains to be studied. We also show that under some conditions—in particular, in the context of pre-established AB memories—IFG may perform integration. These data suggest that for prefrontal regions, as in HPC, the specifics of the learning experience can influence neural representation.

At the whole-brain level, a number of regions exhibit each of the four potential effects (that is, integration, separation, blocked → integration, and intermixed → integration; Supplementary Table 1), highlighting how complementary neural representations might be stored in different brain regions to simultaneously accomplish both specificity and generalizability of memories. Interestingly, the midbrain shows both pattern separation across conditions and a blocked → integration interaction, similar to anterior HPC and posterior MPFC. Prior research has also implicated the midbrain in encoding overlapping associations^26^, demonstrating greater test-phase activation in both HPC and midbrain related to superior ability to generalize across overlapping experiences. Moreover, recent work suggests that dopaminergic midbrain inputs may modulate HPC mechanisms^26^,^54^ and interactions with PFC^55^. For instance, such inputs may mediate the switch between HPC encoding and retrieval^56^ by detecting deviations in the environment from memory-based expectations^54^. In the present study, midbrain signatures may similarly reflect a mismatch response regulating the switch between pattern separation (encoding) and integration via pattern completion (retrieval).

The widespread separation we demonstrate throughout the neocortex may appear to contradict the CLS framework, which proposes integration in neocortical sites. One possibility is that pattern separation is generally preferred throughout the brain on short timescales like that of the present experiment. The CLS framework suggests that the cortex generalizes slowly over many (interleaved) experiences^2^; thus, it may take more experience with overlapping associations or more time for such an integration bias to emerge in the cortex, as memories become strengthened and consolidated. Moreover, it may be the case that blocked and intermixed training differentially promote integration on short (in which blocked experience is superior) and long (in which interleaved experience may be superior^2^) timescales.

Recent work has also used an RS approach to investigate learning-related changes in neural representations following integration^57^. In that study, both MPFC and HPC exhibited integration of related events (animated clips that comprise a narrative). However, the authors did not demonstrate the simultaneous separation of overlapping events, which prominent computational theories^1^,^2^ predict are necessary for the retrieval of episodic details. Moreover, univariate signal in anterior HPC was modulated by the temporal closeness of viewing related and unrelated events, which the authors interpreted as separation of events that were unrelated to the narrative. These conclusions may seem to contradict those put forth in the present study about the role of anterior HPC in memory integration. However, we argue that there are important differences in the paradigms employed that may explain these discrepancies and suggest interpretative caution when considering the prior findings.

First, that study^57^ did not allow for unbiased comparisons between clips from the same versus different narratives, as narrative membership was confounded with temporal proximity in the design. For this reason, it is impossible to determine whether the reported effects reflect integration of specific clips within a narrative or, rather, a common process engaged during viewing of all clips that were part of any narrative. For instance, the increased neural similarity for related clips may reflect a retrieval process—such as recalling verbal labels that describe the narratives. Such a process would not be engaged during the unrelated clips, which could instead elicit a novelty response. This would explain both their anterior HPC univariate findings and the RS differences for related versus unrelated clips. Second, their task encouraged an integration strategy, as participants were told to determine the relationships among clips. This aspect of their design coupled with a slow trial structure would allow for—or even encourage—explicit retrieval strategies similar to the one described above. For these reasons, their results reflect neither shifts in the neural representations of the items themselves, as we demonstrate here (see below), nor the representational strategies intrinsic to HPC and PFC, given the explicit nature of the integration task.

The present approach allows us to assess the dynamics of memory separation and integration directly by overcoming the confounding factors present in this prior study^57^. First, we quantify separation and integration as the learning-related similarity changes for items from the same *relative to those from different* triads. This procedure rules out a process account in our data. Second, fMRI scanning took place while participants viewed individual items rather than multi-item events^36^,^57^, allowing for estimation of item-specific neural patterns. Moreover, our paradigm discouraged the use of intentional strategies. During learning, participants were unaware that they would later need to link A and C items, thus decreasing the likelihood that they would intentionally integrate in preparation for an upcoming test. For this reason, we are able to index the intrinsic representational codes exploited by different brains to encode related events. Moreover, as the neural data were acquired while participants viewed rapid presentations of individual items and made an orthogonal visual decision, our neural representations are unlikely to reflect the active retrieval or suppression of related items.

One possibility is that the changes reported here reflect true shifts in the neural representations of the items themselves. However, perhaps equally likely, similarity increases for A and C item may reflect (automatic) pattern completion during A and C viewing to a common B representation, whereas similarity decreases reflect pattern completion to two different B representations. That is, for a region demonstrating pattern separation, viewing A may lead to (automatic) pattern completion of B_A_ (the representation of B in the context of A), while C pattern completes to B_C_. Such a mechanism would reflect truly pattern-separated event representations that could be individually retrieved and logically recombined to support AC inference. Importantly, either possibility would inform the neural codes that represent related episodes, as pattern completion to a single B representation could not explain the observed simultaneous increases and decreases in A–C similarity. Our findings highlight that, while much of the memory system may pattern separate, integration across related memories via HPC pattern completion can also emerge automatically under certain learning conditions.

Here we provide key support for the idea that the same memory elements may be represented in strikingly different manners across brain regions. These results provide an empirical account for the intuitive notion that memory representations do not come in a single form; rather, a given experience may have multiple representations^58^,^59^, each advantageous in a different scenario. Moreover, consistent with computational accounts, the type of learning experience influences item representations. We demonstrate dissociations across subregions of HPC, MPFC, and IFG, underscoring the importance of considering the functional heterogeneity of these regions.

These findings suggest a theoretical framework in which posterior HPC supports separation of related events in connection with lateral and rostromedial PFC, while anterior HPC and subgenual MFPC integrate across experiences, especially when initial memories are strong. In contrast, experiencing related events in close temporal proximity may lend itself to a pattern separation scheme throughout this network, as weaker overlapping memories are especially prone to interference. The results presented here provide a promising avenue for future computational research, which may incorporate RSA into formal tests of model frameworks.

## Methods

### Participants

Thirty right-handed volunteers (15 women; ages 18–27 years; mean±s.e.m.=21.7±0.5 years) participated in the experiment. This sample size was chosen on the basis of related work in our laboratory^12^,^13^,^21^,^27^. Consent was obtained in accordance with an experimental protocol approved by the Institutional Review Board at the University of Texas at Austin. Participants received monetary compensation for their involvement in the study. Data from a total of four participants were excluded for hardware malfunction (*N*=1), failure to complete the experiment due to illness (*N*=1), instruction error (*N*=1), and low memory performance (*N*=1). Low memory performance was defined as failure to reach above 80% correct on the directly learned (AB and BC) associations as assessed during the post-scan memory test. Data from the remaining 26 participants were included in all analyses (14 women; ages 18–27 years; 21.6±0.5 years).

### Materials

Stimuli consisted of 36 multicoloured novel objects (a subset of which were adapted from a prior study^60^) created using Blender, an open source three-dimensional (3D) animation suite ( www.blender.org). Novel objects were made to appear physically feasible but distinct from real-world objects. We chose novel rather than common objects to avoid stimuli with pre-existing representations.

### Memory task

Novel objects were arranged into 12 ABC triads. ABC triads were presented to participants as overlapping AB and BC pairs, with the B item shared between pairs (Fig. 1a). That is, AB pairs consisted of two novel objects, A and B; the B object was then later paired with a new novel object C to form a BC pair. Overlapping AB and BC pairs were divided into two learning conditions comprising six ABC triads each: blocked and intermixed. In the blocked learning condition, all AB pair presentations occurred before the presentation of any BC pairs (Fig. 1a, pink). In the intermixed learning condition, AB and BC pairs were presented in alternation; that is, for a given ABC triad, the participant first saw AB, then BC, then AB, then BC and so on (Fig. 1a, teal). For both conditions, triads were presented in a pseudo-random order, with the constraint that two pairs from the same triad (multiple presentations of a single AB or BC; or an AB and its corresponding BC) were not presented in immediate succession. The assignment of stimuli to conditions and the order of learning conditions (that is, whether blocked or intermixed learning occurred first) were counterbalanced across participants.

During a study phase (not scanned; Fig. 1a), novel object pairs were presented on the screen for 3.5 s with an interstimulus interval (ISI) of 0.5 s. Each of the 24 AB and BC pairs was presented 12 times. The left/right position of stimuli on the screen was randomized across presentations. Participants were asked to try their best to remember the pairs by creating a visual or verbal story; no overt response was required. Importantly, participants were not made aware of the overlap between AB and BC pairs before beginning the experiment; that is, no instructions were given as to how they should remember the overlapping associations. The study was broken up into two parts (blocked and intermixed learning) of 9.6 min each, with the order counterbalanced across participants. Participants were given the opportunity to take a short break between study portions, if they so wished.

Participants were exposed to single items both immediately before and following study during fMRI scanning (Fig. 1a). The goal of the pre- and post-study exposure phases was to assess the effect of experience on the neural representations of individual A and C items. While prior work has taken a similar approach to demonstrate similarity increases for items that were directly paired in a temporal sequence^34^, here we index changes in similarity for indirectly related items. During this task, participants viewed novel objects on the screen in isolation while performing an orthogonal change-detection task. Trials were 4 s in duration. At the beginning of the trial, a single novel object appeared on screen for 300 ms. At a random time during this 300-ms interval, the superimposed black fixation cross turned either blue or green. Participants were instructed to indicate with a button press whether the cross turned blue or green. Participants had until the end of the 4-s trial to make their response, but were asked to respond as quickly and accurately as possible. These responses were collected solely to ensure attention to the stimuli and were not considered in the analysis.

During each functional run, each of the 36 items was presented in isolation exactly twice. Items were presented in a random order, with the additional constraint that any two items from the same triad (for example, A and C from triad 1) were presented with no fewer than two other items in between them. This was done to ensure that the parameter estimate for the item of interest was not contaminated with lingering activation associated with viewing another item in the same triad. All items were presented once before any item appeared for a second time (that is, both halves of each run contained exactly one presentation of all 36 items). The 72-item trials were randomly intermixed with 24 null fixation trials (4 s long), yielding a total run length of 6.4 min. There were four pre-study exposure scans and four post-study exposure scans. The ordering of trials and scans was identical between the pre- and post-study exposure scans to ensure that pre- and post-study activation measures were not differentially influenced by stimulus presentation order^61^.

Following scanning, it was explained to participants that A and C items could be indirectly related through their common association with a single item, B. After ensuring that participants understood the inference test, a two-alternative forced choice test over the inference (AC) and directly learned (AB, BC) associations was administered (Fig. 1b). All AC inferences were tested before AB and BC pairs to prevent additional learning of the direct associations. Test trials (AC, AB or BC) from the same triad never occurred in immediate succession; the order of trials was otherwise random. A test trial consisted of three items presented on the screen: a cue item at the top and two options on the bottom. For AC and BC test trials, C items served as cues; B items served as cues for AB test trials. Participants were instructed to select which of the bottom two items was associated with the top item. The test was self-paced. Incorrect options (that is, foils) were always familiar items that were members of another triad in the same learning condition. Proportion correct was computed both across learning condition and separately for each learning condition (blocked and intermixed) and test trial (AC, AB and BC) type and averaged across participants. Differences in performance across conditions were assessed using a 3 × 2 repeated measures analysis of variance with test trial type (AC, AB and BC) and learning condition (blocked and intermixed) as within-participant factors.

Before scanning, participants had the opportunity to practice study, test, and exposure tasks. The practice study and test stimuli were novel objects that were not included in the main experiment. The practice pairs were not overlapping, so as to not encourage any strategy in particular before beginning the experiment. The practice exposure task consisted of a single presentation of each of the 36 items used in the main experiment. This was performed to minimize stimulus novelty effects in the scanner.

### MR data acquisition

Imaging data were acquired on a 3.0T Siemens Skyra MRI system. Functional data were collected in 72 oblique axial slices using an echo planar imaging (EPI) sequence, oriented ∼20° off the AC–PC axis (repetition time (TR)=2,000 ms, echo time (TE)=31 ms, flip angle=73; 128 × 128 × 72 matrix, 1.7 mm isotropic voxels, multiband acceleration factor=3, GRAPPA factor=2). Two field maps were collected (TR=589 ms, TE=5 ms/7.46 ms, flip angle=5 degrees; matrix size=128 × 128 × 60; 1.5 × 1.5 × 2 mm voxels) to allow for correction of magnetic field distortions. One was collected before the first functional run (that is, first pre-study exposure) and one before the fifth functional run (that is, first post-study exposure). Two oblique coronal T2-weighted structural images in the same prescription were acquired perpendicular to the main axis of the HPC (TR=13,150 ms, TE=82 ms, 512 × 60 × 512 matrix, 0.4 × 0.4 mm in-plane resolution, 1.5 mm thru-plane resolution, 60 slices, no gap). These images were later co-registered and averaged to generate a mean coronal image for each participant. A T2-weighted structural image in the same prescription as the functional images (that is, coplanar image; TR=15,780 ms, TE=82 ms, 512 × 512 matrix, 0.4 × 0.4 mm in-plane resolution) and a T1-weighted 3D magnetization-prepared rapid gradient echo (MPRAGE) volume (256 × 256 × 192 matrix, 1 mm isotropic voxels) were also collected to facilitate image co-registration, intracranial volume estimation using Freesurfer^62^, and spatial normalization to the MNI template brain.

### fMRI preprocessing

Data were preprocessed and analysed using FSL version 5.0 (FMRIB's Software Library, http://www.fmrib.ox.ac.uk/fsl) and Advanced Normalization Tools (ANTS)^63^. Motion correction was applied to each functional run using MCFLIRT, part of FSL. All functional runs were then registered to a single functional reference run (here, the fourth run) by applying affine transformations calculated in ANTS to each functional time series. Each participant's coplanar, MPRAGE, and mean coronal structural images were registered to their functional data using affine transformations implemented in ANTS. The coplanar image was registered to the functional reference run following field map-based unwarping of the functional data (see below). Transformations were computed for the MPRAGE to the coplanar image and the mean coronal to the MPRAGE. Appropriate transformations were concatenated and applied such that all structural images were moved to functional space and resampled to functional dimensions. For each registration to functional space (coplanar, MPRAGE, and mean coronal), resampling occurred only once. Non-brain structures were removed from the coplanar, MPRAGE, and functional images using BET, part of FSL. Brain extraction for the mean coronal image was performed using the brain mask from the MPRAGE. With the exception of group-level statistics, all analyses were carried out in the native functional space of each participant.

Pre-statistics processing was carried out using FEAT (FMRI Expert Analysis Tool) Version 6.00, part of FSL. The following processing was applied: field map-based EPI unwarping using PRELUDE+FUGUE^64^; grand-mean intensity normalization of the entire four-dimensional (4D) data set by a single multiplicative factor; highpass temporal filtering (Gaussian-weighted least-squares straight line fitting, with sigma=50 s); and spatial smoothing using a Gaussian kernel of 4 mm full-width at half-maximum. The first field map was used to unwarp the pre-study exposure scans; the second field map was used to unwarp the post-study scans. This was done to provide equally optimal unwarping for both scanned phases of the experiment, as any movements during the intervening 20-min study phase may have caused small changes in the magnetic field.

### Estimation of item-level neural patterns

Item-level neural patterns were generated under the assumptions of the general linear model using a modified LS-S approach^65^. Parameter estimate images associated with each of the 36 novel objects were extracted for each run and each participant using custom Python routines. Item presentations were modelled as 0.3-s events and convolved with the canonical (double gamma) haemodynamic response function. The two presentations of each item were modelled as a single regressor. Motion parameters calculated during the motion correction step and their temporal derivatives were added as additional confound regressors. Framewise displacement and DVARS, two measures of framewise data quality, were also added to the model as regressors of no interest^21^,^66^. Additional regressors were created for each time point in which motion exceeded a threshold of both 0.5 mm for framewise displacement and 0.5% change in BOLD signal for DVARS (plus one time point before and two time points after)^66^. Temporal filtering was applied to the model. This process resulted in one statistics image for each of 36 items in each of eight runs (for a total of 288 images per participant).

### RSA searchlight

Searchlight RSA was carried out using the PyMVPA toolbox^67^ and custom Python routines. This approach was used to identify voxels showing one of the four hypothesized learning-related signatures in A-C RS (Fig. 2): (1) integration for both learning conditions; (2) separation for both learning conditions; (3) interaction, with blocked → integration (blocked>intermixed × within>across-triad similarity); and (4) interaction, with intermixed → integration (intermixed>blocked × within>across-triad similarity). All analyses were limited to those A and C items for which the corresponding AC inference was correct during the test.

We searched for each of these four effects using searchlights restricted to voxels in three *a priori* anatomical ROIs: bilateral HPC, MPFC, and IFG. We also ran searchlight analyses across the whole brain, unrestricted to any particular region. ROIs were defined on custom coronal (HPC) and MNI (MPFC and IFG) template brains (Supplementary Methods) and reverse normalized to each participant's functional space to carry out the searchlight analyses. Group ROIs were used to ensure that the spatially normalized statistical maps (see below) would be overlapping, thereby enabling comparisons across participants.

A spherical searchlight (radius=3 voxels; volume=123 voxels, except along ROI boundaries) was swept across each anatomical ROI. For each sphere (that is, centred on every voxel in the ROI), a statistic of interest was calculated from the item-level pairwise comparisons of activation patterns as follows. Pairs of item-level activation patterns from pre- and post-study exposure phases were compared separately using Pearson's correlation, transformed to Fisher's *z* and subtracted, resulting in a post–pre similarity score for each pair of items (hereafter, change in (Δ) similarity). Changes in pairwise similarity for comparisons of interest were then averaged to yield mean within- (that is, the average of all within-triad (for example, A1–C1) comparisons) and across-triad (that is, the average of all across-triad (for example, A1–C2) comparisons) Δ similarities for blocked and intermixed learning. Importantly, for both within- and across-triad Δ similarity calculations, comparisons were limited to pairs of activation patterns extracted from different runs. This was performed to ensure independence of the activation patterns going into Δ similarity calculations, thus preserving the false-positive rate^61^. Across-triad comparisons were additionally limited to items from the same learning condition (for example, a blocked A item would never be compared with an intermixed C item; Fig. 2, white cells).

For each sphere in the searchlight, contrasts representing the four effects of interest were computed (Fig. 2, inset barcharts) using the mean Δ similarities as follows: (1) integration, blocked within–blocked across+intermixed within–intermixed across; (2) separation, blocked across−blocked within+intermixed across–intermixed within; (3) blocked → integration interaction, blocked within–blocked across+intermixed across–intermixed within; (4) intermixed → integration interaction, intermixed within–intermixed across+blocked across–blocked within. Contrasts were converted to *P* values by comparing the observed contrast value to a permutation-based null distribution. We generated null distributions by randomly shuffling within- and across-triad Δ similarity correlation values (within learning condition; equivalent to shuffling within- and across-triad comparison labels) and re-computing the statistic of interest for each of 1,000 iterations. This *P* value was assigned to the centre voxel of the current sphere; the sphere was then shifted and the entire procedure repeated. Conducting the searchlight RSA for each participant resulted in four *P* value maps for each of the three anatomical ROIs and for the whole brain.

Each participant's voxelwise *P* values were converted to *z*-statistics (allowing for both positive and negative values) and the resulting maps were warped to the MNI template (resampled to the functional dimensions of the present study, 1.7 mm isotropic) by applying nonlinear transformations computed previously using ANTS^63^. *Z*-statistics were then combined across the group using nonparametric one-sample *t*-tests implemented in Randomise^68^, part of FSL. For the whole-brain analysis, statistics images were first masked to exclude white matter. We then applied a primary voxelwise threshold of *P*<0.01 (uncorrected) to all group statistics images to identify those voxels surpassing this initial *P* value threshold. Significant cluster sizes within each anatomical ROI (HPC, MPFC, and IFG) and across the whole brain were determined using Monte Carlo simulations implemented in 3dClustSim, part of AFNI^69^. Cluster sizes that occurred with a probability of less than 0.05 across 2,000 simulations were considered statistically significant. This was performed separately for each ROI and for the whole brain, restricted to grey matter. Follow-up analyses were also conducted in which we determined the within- versus across-triad Δ similarities for blocked and intermixed learning conditions separately (Supplementary Methods).

### HPC volume-Δ similarity analysis

We next investigated the relationship between measures of HPC structure and coding strategy. Specifically, we related the volume of HPC subregions (head, body, and tail) to the neural evidence for integration and separation from the RSA described above. We tested the hypothesis that HPC head volume would relate to the degree of integration, particularly for blocked triads.

We extracted bilateral HPC head, body, and tail volumes for each participant. To account for differences in overall head size (approximated by intracranial volume, derived from Freesurfer^62^) across participants, HPC volumes were adjusted using an analysis of covariance approach^70^. As our neural similarity measures, we extracted average *z*-statistics representing integration for blocked and separation for intermixed conditions for each participant across the whole anatomical HPC ROI in template space. We then related HPC subregion volumes to neural similarity using Pearson correlation. We also performed two multiple regressions on *z*-scores, with HPC head, body, and tail volumes as independent variables and neural evidence for integration in the blocked condition and separation in the intermixed condition, respectively, as the dependent measures.

## Additional information

**How to cite this article:** Schlichting, M. L. *et al.* Learning-related representational changes reveal dissociable integration and separation signatures in the hippocampus and prefrontal cortex. *Nat. Commun.* 6:8151 doi: 10.1038/ncomms9151 (2015).

## Supplementary Material

Supplementary Information Supplementary Figures 1-3, Supplementary Table 1, Supplementary Methods and Supplementary References
