# Supplementary material for: Learning-related representational changes reveal dissociable integration and separation signatures in the hippocampus and prefrontal cortex
Source: Nat Commun. 2015 Aug 25;6:8151. doi: 10.1038/ncomms9151 (PMC4560815; doi:10.1038/ncomms9151)
Supplement: Supplementary Information — Supplementary Figures 1-3, Supplementary Table 1, Supplementary Methods and Supplementary References [file ncomms9151-s1.pdf]

SUPPLEMENTARY FIGURES

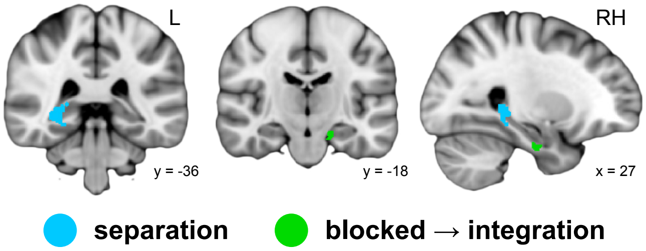

Supplementary Figure 1. Results of Conjunction Analysis in HPC

Voxels showing main effect of separation (blue) reached individual mapwise thresholds for contrasts indexing both (1) separation for blocked triads and (2) separation for intermixed triads. Voxels showing blocked → integration interaction (green) reached individual mapwise thresholds for contrasts indexing both (1) integration for blocked triads and (2) separation for intermixed triads. Clusters are displayed on the 1mm MNI template brain with a conjunction threshold of $p < 0.005$, uncorrected and a cluster extent threshold of 10 contiguous voxels. Coordinates are in millimeters. N = 26 participants.

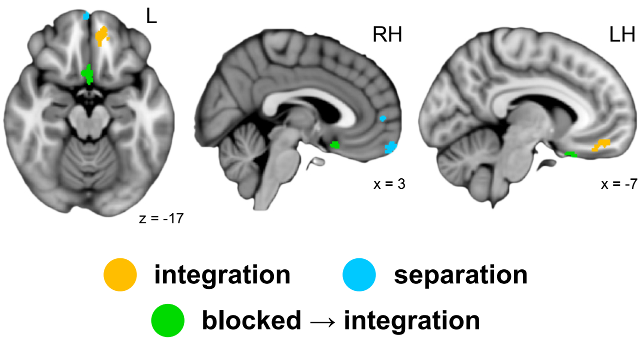

Supplementary Figure 2. Results of Conjunction Analysis in MPFC

Separation and blocked → integration voxels are as described in **Supplementary Fig. 1**. Voxels showing main effect of integration (orange) reached individual mapwise thresholds for contrasts indexing both (1) integration for blocked triads and (2) integration for intermixed triads. Clusters are displayed on the 1mm MNI template brain with a conjunction threshold of $p < 0.005$, uncorrected and a cluster extent threshold of 10 contiguous voxels. Coordinates are in millimeters. N = 26 participants.

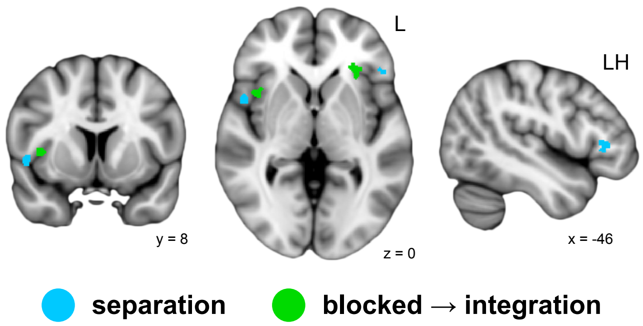

Supplementary Figure 3. Results of Conjunction Analysis in IFG

Separation and blocked → integration voxels are as described in **Supplementary Fig. 1**. Clusters are displayed on the 1mm MNI template brain with a conjunction threshold of $p < 0.005$, uncorrected and a cluster extent threshold of 10 contiguous voxels. Coordinates are in millimeters. N = 26 participants.

SUPPLEMENTARY TABLE

Region	*Hemisphere*	*N vox*[a]	t_{25}	*X*[b]	*Y*[b]	*Z*[b]
Integration						
Middle frontal gyrus	R	79	2.3	26	8	51
Frontal pole	L	72	2.54	-17	61	6
Separation						
Fusiform/lingual gyrus	B	484	1.92	10	-83	-18
Inferior temporal/fusiform gyrus	R	342	2.09	48	-58	-24
Temporal pole	R	320	3.39	35	16	-36
Pons	B	320	2.72	-10	-28	-42
Hippocampus, posterior	R	261	2.08	19	-32	-3
Midbrain/cerebellum	R	261	2.71	19	-27	-27
Temporal pole/planum porale	R	240	2.87	48	4	-7
Insula/pars opercularis	L	223	1.53	-41	-2	1
Lateral occipital cortex	R	204	2.93	49	-74	6
Supramarginal gyrus (IPL)	L	161	2.89	-54	-37	36
Precentral gyrus	B	132	3.09	0	-27	54
Midbrain/thalamus	L	123	2.94	-4	-31	0
Superior temporal gyrus	L	111	4.03	-64	-29	3
Precuneus	L	106	3.7	-7	-53	43
Midbrain/parahippocampal cortex	L	96	2.65	-13	-26	-18
Nucleus accumbens	B	90	3.95	1	3	-7
Lingual gyrus	L	87	4.13	-17	-43	-6
Intracalcarine sulcus	R	76	3.32	16	-71	6
Superior frontal gyrus	R	69	3.39	8	19	66
Medial frontal pole	R	69	2.72	1	60	-20
Anterior cingulate gyrus	R	67	4.55	12	35	21
Blocked → Integration (blocked > intermixed × integration > separation)						
Midbrain	R	328	3.52	6	-21	-19
Lingual gyrus	R	229	3.02	14	-47	-8
Insula	L	205	3.6	-44	-11	1
Subgenual cortex	B	181	2.59	1	17	-19
Planum temporale	L	124	3.91	-37	-34	19
Middle frontal gyrus	L	98	4.51	-35	32	37
Putamen	R	77	4.24	22	13	-6
Lingual gyrus	B	70	2.72	4	-74	-9
Intermixed → Integration (intermixed > blocked × integration > separation)						
Pre/Postcentral gyrus	R	92	3.29	36	-24	68
Precuneus	R	83	2.04	11	-55	21
Superior frontal gyrus	L	72	3.11	-18	45	41
Precuneus	R	72	3.64	9	-62	38
Middle temporal gyrus	R	70	2.34	54	-2	-30

[a]1.7 mm isotropic voxels
[b]MNI coordinates, rounded to the nearest mm

Supplementary Table 1. Whole Brain RSA Searchlight Results. Values reflect cluster center of gravity (COG). N = 26 participants.

SUPPLEMENTARY METHODS

Region of interest definition. Anatomical ROIs were used to restrict RSA searchlight analyses. HPC was manually demarcated on a custom template generated from the mean coronal images; MPFC and IFG ROIs were created on the MNI template brain. Different procedures were used to define HPC and PFC ROIs to account for the high variability in hippocampal anatomy across individuals. These procedures are described in detail below.

A custom coronal template was generated using ANTS[1]. The T2-weighted mean coronal images from a subset of ten participants with canonical hippocampi were selected for template generation. A bilateral HPC ROI was delineated by hand on the coronal template using established guidelines[2]. HPC was further segmented into anterior (head) and posterior (body and tail) subregions for volume analysis using anatomical landmarks as follows. The posterior boundary of the HPC head was the last slice on which the uncal apex was visible[3,4]. The anterior boundary of the HPC tail was the first slice on which the fornix became visibly separated from the HPC[5]. Nonlinear transformations were calculated to normalize each participant's mean coronal image to the coronal template. The inverse of these transformations were then concatenated with the coronal to functional (affine) transformation and applied to the HPC ROIs. This process resulted in bilateral HPC head, HPC body, HPC tail, and overall HPC ROIs in the native functional space of each participant.

Prefrontal ROIs were defined on the MNI template brain. MPFC was delineated by hand to approximate all cytoarchitectonic subdivisions thought to be part of the

medial prefrontal network, which is most related to limbic structures such as the HPC[6,7]. IFG was created by summing subdivisions pars opercularis, pars triangularis, and pars orbitalis derived from Freesurfer[8]. ROIs were then inflated to allow for variability in neocortical anatomy across participants and reverse normalized to each participant's native functional space.

Follow-up simple contrasts and conjunction analyses. Because the RSA searchlights described in **Results** interrogated the brain for main effects and interactions across learning conditions, one possibility is that any observed effects might be driven by significant within- versus across-triad differences in one learning condition but not the other. To assess this possibility, we performed follow-up analyses in which we determined the within- versus across-triad Δ similarities for blocked and intermixed learning conditions separately.

Searchlights were run for each of the following learning-changes in A-C RS: (1) integration for blocked triads; (2) integration for intermixed triads; (3) separation for blocked triads; (4) separation for intermixed triads. We searched for these patterns within HPC, MPFC and IFG. Importantly, search areas were not restricted to regions previously identified in the main searchlight analyses; rather, they were run across the entire extent of each anatomical ROI. With the exception of the contrast values calculated within each sphere, searchlight analyses at the individual participant level were identical to those described previously (**Methods**). Contrasts were calculated in the following manner: (1) integration for blocked triads, blocked within – blocked across; (2) integration for intermixed triads, intermixed within – intermixed across; (3) separation

for blocked triads, blocked across – blocked within; (4) separation for intermixed triads, intermixed across – intermixed within. Permutation tests were performed as described previously, yielding four p-value maps for each of the three ROIs for each participant. Each participant's voxelwise p-value maps were converted to z-statistics and the resulting images were warped to the 1.7 mm isotropic MNI template using ANTS[1].

Simple contrasts functional ROI analysis. We then interrogated individual within- versus across-triad differences within each cluster identified as showing one of the four predicted patterns in the main searchlight analysis. Average z-statistics representing the integration or separation effects for each learning condition separately were extracted and compared with zero using a bootstrapping approach. Participants were resampled with replacement and the average z-statistic across the simulated group was computed for each ROI on each of 100,000 iterations. P-values were determined as the proportion of iterations on which the group average was less than zero.

Conjunction analysis. To ensure that our effects were not inflated by limiting within- versus across-triad comparisons to only those voxels identified in the main contrasts, we also performed a follow-up conjunction analysis that investigated the overlap among contrasts of interest in our anatomical ROIs (HPC, MPFC, and IFG). As this analysis reduced the number of triads by approximately half (depending on AC performance), it should be noted that this analysis represents a substantial reduction in statistical power and is intended as complementary to the main searchlight analyses.

Non-parametric one-sample t-tests were carried out at the group level as described previously. We were interested in identifying voxels showing integration for both blocked and intermixed training (i.e., the overlap of contrasts 1 and 2); separation

for both blocked and intermixed training (contrasts 3 and 4); and a blocked → integration interaction (contrasts 1 and 4), mirroring the main contrasts. For each contrast image of interest, we applied a primary voxelwise threshold of $p < \sqrt{0.005}$ (uncorrected) and binarized the resulting maps. Because we were interested in determining the overlap among pairs of maps, this resulted in a conjunction threshold of $p < 0.005$, uncorrected. Overlapping voxels were those that reached the $p < \sqrt{0.005}$ threshold in both underlying contrasts of interest and are displayed with an extent threshold of 10 contiguous voxels in **Supplementary Figs. 1-3**. This approach produced similar results to the main analyses, suggesting that the observed effects were driven by significant within- versus across-triad differences in each of the underlying learning conditions.

Across-triad change functional ROI analysis. Because our main searchlight analyses indexed significant integration and separation as change for within- relative to across-triad comparisons, it is not clear whether items from different triads in the same learning condition became significantly more (or less) similar to one another following learning. One possibility is that all items from a given condition became more or less similar to one another (depending on the region), with within-triad comparisons changing the most; alternatively, it might be the case that across-triad similarities remained the same, with only the within-triad similarities changing as a function of learning. To assess these possibilities, we performed a follow-up analysis that quantified the degree of change in neural similarity for across-triad comparisons.

Searchlights were run within anatomical HPC, MPFC, and IFG to test for

significant increases or decreases in across-triad RS, separately for blocked and intermixed conditions. Within each sphere of the searchlight, a difference statistic representing the change in across-triad similarity from pre- to post-study was calculated and compared against a null distribution. Null distributions were computed by shuffling whether similarity values came from the pre- or post-study scan (within a given comparison) and re-computing the difference statistic for each of 1,000 iterations. Participant-level voxelwise p-value maps were converted to z-statistics warped to the 1.7 mm isotropic MNI template using ANTS[1] as described previously.

We then interrogated each of the clusters identified as showing one of the four predicted patterns in the main searchlight analysis for signs of change for across-triad comparisons. Average z-statistics representing the change in across-triad similarity for each learning condition separately were extracted and compared with zero (using a two-tailed test, allowing for both similarity increases and decreases) using a bootstrapping approach. Correction for multiple comparisons was performed using Bonferroni correction, yielding a critical p-value of 0.005 (0.05/11 clusters).

Control analyses. As encoding order (i.e., whether blocked or intermixed learning occurred first) was counterbalanced across participants, individuals had substantial differences in learning experience that might impact their behavioral performance and/or neural coding. Accordingly, we performed control analyses to test for effects of encoding order on behavioral and neural measures of interest.

<u>Effects of encoding order on behavior.</u> We first interrogated whether behavioral performance was significantly modulated by encoding order. We performed a 3 × 2 × 2

mixed ANOVA with test trial type (AC, AB, BC) and learning condition (blocked, intermixed) as within-participant factors and encoding order as the between participants factor. Memory performance (proportion correct) served as the dependent measure. Order of blocked versus intermixed learning (i.e., encoding order) did not significantly affect behavior (main effect of order and two- and three-way interactions; all $F < 1.91$, all $p > 0.180$).

Effects of encoding order on Δ RS. Average z-statistics were extracted for each participant across every cluster identified in the main searchlight analyses within our *a priori* anatomical ROIs. These z-statistics represented the degree to which each participant exhibited the effect of interest (i.e., the effect that was significant in that particular cluster). We tested whether these values differed as a function of encoding order using a two-sample t-test. Correction for multiple comparisons was performed as described above, yielding a critical p-value of 0.005. Encoding order did not significantly modulate changes in neural pattern similarities in any region (Bonferroni-corrected α threshold for significance < 0.005; HPC searchlight: all $t_{24} < 2.06$, all $p > 0.051$; MPFC searchlight: all $|t_{24}| < 2.63$, all $p > 0.014$; IFG searchlight: all $|t_{24}| < 2.14$, all $p > 0.043$).

Effects of encoding order on HPC volume-Δ RS relationship. We also performed one-way analyses of covariance (ANCOVA) to interrogate whether the observed relationships between anterior HPC (head) volume and neural similarity measures differed significantly as a function of encoding order. HPC subregion volumes (head, body, and tail) served as the predictor variables; neural similarity (integration in the blocked condition, and separation in the intermixed condition) served as the response. Encoding order was the grouping variable. Correction for multiple comparisons was

performed using Bonferroni correction, yielding a critical p-value of 0.017 (0.05/3 subregions). Encoding order did not significantly impact the relationships between HPC head volumes and integration in the blocked condition (main effect and interaction; both $F_{1,22} < 1.80$, both $p > 0.194$) or separation in the intermixed condition (both $F_{1,22} < 0.49$, both $p > 0.490$). This was also true for HPC body (main effects and interactions; all $F_{1,22} < 2.97$, all $p > 0.099$) and tail (all $F_{1,22} < 2.52$, all $p > 0.127$) volumes.

SUPPLEMENTARY REFERENCES

1. Avants, B. B. *et al.* A reproducible evaluation of ANTs similarity metric performance in brain image registration. *Neuroimage* **54,** 2033–44 (2011).

2. Amaral, D. G. & Insausti, R. in *Hum. Nerv. Syst.* (Paxinos, G.) 711–755 (Academic Press, 1990).

3. Weiss, A. P., Dewitt, I., Goff, D., Ditman, T. & Heckers, S. Anterior and posterior hippocampal volumes in schizophrenia. *Schizophr. Res.* **73,** 103–112 (2005).

4. Poppenk, J. & Moscovitch, M. A hippocampal marker of recollection memory ability among healthy young adults: contributions of posterior and anterior segments. *Neuron* **72,** 931–937 (2011).

5. Watson, C. *et al.* Anatomic basis of amygdaloid and hippocampal volume measurement by magnetic resonance imaging. *Neurology* **42,** 1743–1750 (1992).

6. Ongür, D., Ferry, A. T. & Price, J. L. Architectonic subdivision of the human orbital and medial prefrontal cortex. *J. Comp. Neurol.* **460,** 425–49 (2003).

7. Price, J. L. & Drevets, W. C. Neurocircuitry of mood disorders. *Neuropsychopharmacology* **35,** 192–216 (2009).

8. Desikan, R. S. *et al.* An automated labeling system for subdividing the human cerebral cortex on MRI scans into gyral based regions of interest. *Neuroimage* **31,** 968–80 (2006).
